# Recent Advances in the Evaluation of Serological Assays for the Diagnosis of SARS-CoV-2 Infection and COVID-19

**DOI:** 10.3389/fpubh.2020.620222

**Published:** 2021-02-18

**Authors:** Angela Chiereghin, Rocco Maurizio Zagari, Silvia Galli, Alessandra Moroni, Liliana Gabrielli, Simona Venturoli, Isabella Bon, Giada Rossini, Ilaria Maria Saracino, Matteo Pavoni, Silvia Lafratta, Alessandro Deni, Silvia Felici, Michele Borghi, Luca Guerra, Luigi Raumer, Vittorio Lodi, Pierluigi Viale, Luciano Attard, Tiziana Lazzarotto, IRCCS St. Orsola Polyclinic of Bologna COVID-19 Research Team

**Affiliations:** ^1^Microbiology Unit, Department of Specialized, Experimental, and Diagnostic Medicine, Istituto di Ricovero e Cura a Carattere Scientifico St. Orsola Polyclinic, University of Bologna, Bologna, Italy; ^2^Department of Medical and Surgical Sciences, Istituto di Ricovero e Cura a Carattere Scientifico St. Orsola Polyclinic, University of Bologna, Bologna, Italy; ^3^Microbiology Unit, Istituto di Ricovero e Cura a Carattere Scientifico St. Orsola Polyclinic, University of Bologna, Bologna, Italy; ^4^Infectious Diseases Unit, Department of Medical and Surgical Sciences, Istituto di Ricovero e Cura a Carattere Scientifico St. Orsola Polyclinic and Azienda Unita' Sanitaria Locale Bologna, Bologna, Italy; ^5^Occupational Health Unit, Istituto di Ricovero e Cura a Carattere Scientifico St. Orsola Polyclinic, University of Bologna, Bologna, Italy; ^6^Infectious Diseases Unit, Department of Medical and Surgical Sciences, Istituto di Ricovero e Cura a Carattere Scientifico St. Orsola Polyclinic, University of Bologna, Bologna, Italy

**Keywords:** SARS-CoV-2 infection, COVID-19, SARS-CoV-2 RT-PCR, SARS-CoV-2-specific antibodies, LFIA, CLIA, ECLIA and ELISA, sensitivity and specificity

## Abstract

**Introduction:** Few data on the diagnostic performance of serological tests for severe acute respiratory syndrome coronavirus 2 (SARS-CoV-2) infection are currently available. We evaluated sensitivity and specificity of five different widely used commercial serological assays for the detection of SARS-CoV-2–specific IgG, IgM, and IgA antibodies using reverse transcriptase-PCR assay in nasopharyngeal swab as reference standard test.

**Methods:** A total of 337 plasma samples collected in the period April–June 2020 from SARS-CoV-2 RT-PCR positive (*n* = 207) and negative (*n* = 130) subjects were investigated by one point-of-care lateral flow immunochromatographic assay (LFIA IgG and IgM, Technogenetics) and four fully automated assays: two chemiluminescence immunoassays (CLIA-iFlash IgG and IgM, Shenzhen YHLO Biotech and CLIA-LIAISON^®^ XL IgG, DiaSorin), one electrochemiluminescence immunoassay (ECLIA-Elecsys^®^ total predominant IgG, Roche), and one enzyme-linked immunosorbent assay (ELISA IgA, Euroimmune).

**Results:** The overall sensitivity of all IgG serological assays was >80% and the specificity was >97%. The sensitivity of IgG assays was lower within 2 weeks from the onset of symptoms ranging from 70.8 to 80%. The LFIA and CLIA-iFlash IgM showed an overall low sensitivity of 47.6 and 54.6%, while the specificity was 98.5 and 96.2%, respectively. The ELISA IgA yielded a sensitivity of 84.3% and specificity of 81.7%. However, the ELISA IgA result was indeterminate in 11.7% of cases.

**Conclusions:** IgG serological assays seem to be a reliable tool for the retrospective diagnosis of SARS-CoV-2 infection. IgM assays seem to have a low sensitivity and IgA assay is limited by a substantial rate of indeterminate results.

## Introduction

Since emerging in late December 2019 in Wuhan, China, the novel coronavirus severe acute respiratory syndrome coronavirus 2 (SARS-CoV-2) has spread rapidly worldwide resulting in a pandemic (1). According to the World Health Organization, as of October 20, 2020, more than 40,000,000 laboratory-confirmed cases and over 1,000,000 deaths have been globally reported (2). Currently, the laboratory confirmation of possible and probable cases of coronavirus disease 2019 (COVID-19) is based on the detection of the viral genome in respiratory tract specimens by nucleic acid amplification tests such as the real-time reverse transcriptase (RT)-PCR assay (3). However, the diagnostic accuracy of the molecular testing may be affected by some factors such as the time window of viral replication, the magnitude of viral load at the site of sample collection, and the quality of sample collection (4). Concerning the serological testing, though clinical utility is currently unclear (5), it is known that validated serological assays have important application areas, for instance for patient contact tracing and for epidemiological studies (6). In this regard, although many serological assays have been rapidly developed and made commercially available during this pandemic, only limited clinical validations considering different groups of subjects such as those who developed an asymptomatic infection or with probable COVID-19 as well as the timing of sample collection in relation to symptoms onset has been currently performed (7). The aim of this study was to evaluate the diagnostic performance of five different widely used commercial serological assays for the detection of SARS-CoV-2–specific IgG, IgM, and IgA antibodies using the US Center for Disease Prevention and Control SARS-CoV-2 real-time RT-PCR in nasopharyngeal swab as reference standard test. A secondary aim was to assess the agreement between different serological assays by class of immunoglobulin detected (IgG or IgM).

## Materials and Methods

### Study Design

This is a retrospective case-control study evaluating the sensitivity and specificity of a point-of-care (POC) lateral flow immunochromatographic assay (LFIA) and four fully automated assays, two chemiluminescence immunoassays (CLIAs), an electrochemiluminescence immunoassay (ECLIA), and an enzyme-linked immunosorbent assay (ELISA), for the detection of SARS-CoV-2–specific IgG, IgM, and IgA antibodies in blood samples.

### Study Sample

Residual frozen plasma samples from asymptomatic and symptomatic individuals with positive or negative SARS-CoV-2 RNA nasopharyngeal swab were collected from April to June 2020 during routine serological investigations performed at the Operative Unit of Clinical Microbiology of the IRCCS St. Orsola Polyclinic, University of Bologna, Italy. Asymptomatic subjects underwent molecular testing for SARS-CoV-2 infection given that they met at least one of the two epidemiological criteria for coronavirus disease 2019 of the European Center for Disease Prevention and Control (8). We excluded subjects with a negative SARS-CoV-2 RT-PCR who met clinical and epidemiological or imaging criteria of COVID-19 (probable COVID-19–positive patients) (8). The study was approved by the Ethical Committee of the University of Bologna.

### Serological Assays

Blood samples were collected in ethylenediamine tetraacetic acid–anticoagulated tubes and plasma sample leftovers were prospectively stored at −80°C until testing. The tests' procedures and the interpretation of results adopted were reported in the manufacturer instructions for all the assays. The evaluation of serological assays was simultaneous.

### Qualitative Detection of SARS-CoV-2 IgG and IgM

The nCOVID-19 IgG and IgM POCT (Technogenetics S.r.l., Milan, Italy) LFIA and the SARS-CoV-2 IgM and IgG CLIA kits (Shenzhen YHLO Biotech Co., Ltd., China) were used; these assays are CE marked. Briefly, the POC test for the rapid detection of both IgG and IgM antibodies in human serum, plasma, and whole blood samples was performed in laboratory by testing plasma samples. The assay had a turnaround time (TAT) of 15 min; results were evaluated independently by two different investigators and faint banding for IgG and/or IgM was considered positive. The assay detects antibodies to the nucleocapsid protein of SARS-CoV-2. The CLIA assays (hereinafter named CLIA-iFlash) were performed on the iFlash3000 CLIA analyzer (Shenzhen YHLO Biotech Co., Ltd., China); these are high-throughput assays with an estimated TAT of 40 min per sample. The amount of SARS-CoV-2 IgM or IgG in the serum/plasma sample is in proportion to the relative light unit (RLU) measured by the CLIA analyzer that automatically calculates the antibody concentration (in arbitrary units (AU)/ml) on the basis of the RLU and the calibration curve. The cut-off value for reactivity (positivity) is equal to 10.0 AU/mL for both IgG and IgM. The magnetic beads of these assays are coated with recombinant antigens representing SARS-CoV-2 nucleocapsid protein and spike protein.

### Quantitative Detection of SARS-CoV-2 IgG

The LIAISON^®^ SARS-CoV-2 S1/S2 IgG CLIA assay (hereinafter named CLIA-LIAISON^®^ XL; DiaSorin S.p.A., Saluggia, Italy) performed on the LIAISON^®^ XL Analyzer (DiaSorin) was used. It is a high-throughput assay with an estimated TAT of 40 min per sample. The assay is CE marked and in late April 2020, received the Food and Drug Administration's Emergency Use Authorization (EUA). Antibody concentration in serum/plasma sample, expressed as AU/ml, was automatically calculated by the analyzer on the basis of the RLU and the calibration curve. The cut-off value for a positive result is equal to 15 AU/ml. The magnetic beads of the assay are coated with recombinant antigens representing the S1 and S2 subunits of the spike protein of SARS-CoV-2. Given the assay's target, potential neutralizing antibodies could be detected. In this regard, some authors showed that this assay provided the detection of neutralizing antibodies with 94.4% positive agreement and 97.8% negative agreement to plaque reduction neutralization test (9).

### Qualitative Detection of SARS-CoV-2 Total (Predominantly IgG) Antibodies

The Elecsys^®^ Anti-SARS-CoV-2 ECLIA assay (Roche Diagnostics AG, Rotkreuz, Switzerland) performed on the cobas e 801 analyzer (Roche Diagnostics) was used. This is a high-throughput assay with an estimated TAT of 20 min per sample. The assay is CE marked and at the beginning of May 2020, received the Food and Drug Administration's EUA. Results [in cut-off index (COI)] are determined automatically by the analyzer's software that compares the electrochemiluminescence signal obtained from the reaction product of the serum/plasma sample with the signal of the cut-off value previously obtained by calibration. The cut-off value for reactivity (positivity) is equal to 1.0 COI. The assay uses a recombinant protein representing the nucleocapsid antigen, and its format favors the preferential detection of late, mature, and high affinity antibodies. Therefore, despite that this assay detects all classes of immunoglobulin (IgA, IgM, and IgG), it detects predominantly IgG (10).

### Semiquantitative Detection of SARS-CoV-2 IgA

The Anti-SARS-CoV-2 IgA ELISA assay (Euroimmun Medizinische Labordiagnostika, Lübeck, Germany) performed on EUROIMMUN Analyzer I was used. This is a midvolume assay with an estimated TAT of 4 h per 96-well plate; the assay is CE marked. The results are expressed as a ratio between the extinction of the serum/plasma sample, and the calibrator that is automatically calculated by the analyzer. A ratio ≥0.8 to <1.1 identify an equivocal (indeterminate) result; a ratio >1.1 identifies a positive result. The assay uses a recombinant protein representing the S1 subunit of the spike protein of SARS-CoV-2.

### Statistical Analysis

Sensitivity and specificity with 95% confidence interval (CI) of each serological assay were calculated using 2 × 2 tables. Sensitivity and specificity are, respectively, the percentage of subjects with positive and negative SARS-CoV-2 RT-PCR correctly identified by serological assay. The accuracy of each test, that is the percentage of individuals for whom both the serological test and reference standard give the same result, was quantified by receiver operating characteristic (ROC) analysis. Plasma samples with indeterminate results by ELISA IgA were excluded from sensitivity and specificity analyses of this test. Sensitivity and specificity of serological assays was also assessed separately in asymptomatic and symptomatic subjects and by the time elapsed from the onset of symptoms and blood collection (<14 vs. >14 days). We assessed the agreement between serological assays by class of immunoglobulin detected (IgG or IgM) using Kappa statistic. Continuous variables were described using mean and standard deviation (SD). A *p* < 0.05 was considered statistically significant. All statistical analyses were performed using STATA version 15 (StataCorp, College Station, TX, USA).

## Results

During the study period, plasma samples from 361 subjects were collected. Of these, 24 symptomatic subjects with a negative SARS-CoV-2 RT-PCR were excluded as they were probable COVID-19–positive patients. A total of 337 subjects [mean age 59.3, SD 23.8; males: 158 (46.9%)], 284 with symptoms and 53 without symptoms, were included in the study. Of these, 207 were RT-PCR positive (188 with symptoms) and 130 RT-PCR negative (96 with symptoms). Of the RT-PCR–positive subjects, one was not tested by the LFIA IgG and IgM and four by ELISA IgA due to insufficient sample volume.

### Diagnostic Performance

Of the 202 SARS-CoV-2 RT-PCR–positive subjects who underwent all the serological assays, only 17 (8.4%) resulted negative for IgG, IgM, or IgA.

Table 1 shows the sensitivity and specificity of each serological assay. The overall sensitivity of all IgG serological assays was >80% and the specificity was >95%. In particular, the overall sensitivity of IgG serological assays ranged from 81.6% (95% CI, 75.7–86.7) with CLIA-LIAISON^®^ XL to 89.9% (95% CI, 84.9–93.6) with CLIA-iFlash, and the specificity from 97.7% (95% CI, 93.4–99.5) with CLIA-LIAISON^®^ XL to 100% (95% CI, 97.2–100) with ECLIA-Elecsys^®^. The overall sensitivity of IgM serological tests was very low being 47.6% (95% CI, 40.6–54.6) and 54.6% (95% CI, 47.5–61.5) with LFIA and CLIA-iFlash, respectively, while the specificity was 98.5% (95% CI, 94.6–99.8) and 96.2% (95% CI, 91.3–98.7).

**Table 1:** Overall sensitivity and specificity of the serological assays for the diagnosis of SARS-CoV-2 infection using RT-PCR as reference standard.

**Serological assays**			**No. of** **samples**	**RT-PCR** **positive**	**RT-PCR** **negative**	**True** **positive**	**False** **positive**	**True** **negative**	**False** **negative**	**Sensitivity %** **(95% CI)**	**Specificity %** **(95% CI)**
IgG	LFIA	POCT	336	206	130	173	1	129	33	84.0 (78.2–88.7)	99.2 (95.8–100)
	CLIA	iFlash	337	207	130	186	2	128	21	89.9 (84.9–93.6)	98.5 (94.6–99.8)
		LIAISON^®^ XL	337	207	130	169	3	127	38	81.6 (75.7–86.7)	97.7 (93.4–99.5)
	ECLIA	Elecsys^®^	337	207	130	179	0	130	28	86.5 (81.0–90.8)	100 (97.2–100)
IgM	LFIA	POCT	336	206	130	98	2	128	108	47.6 (40.6–54.6)	98.5 (94.6–99.8)
	CLIA	iFlash	337	207	130	113	5	125	94	54.6 (47.5–61.5)	96.2 (91.3–98.7)
IgA	ELISA	Euroimmune I	294	185	109	156	20	89	29	84.3 (78.3–89.2)	81.7 (73.1–88.4)

*SARS-CoV-2, severe acute respiratory syndrome coronavirus 2; RT-PCR, reverse transcriptase-PCR; CI, confidence interval; LFIA, lateral flow immunochromatographic assay; POCT, point-of-care test; CLIA, chemiluminescence immunoassays; ECLIA, electrochemiluminescence immunoassay; ELISA, enzyme-linked immunosorbent assay*.

**Figure 1:**
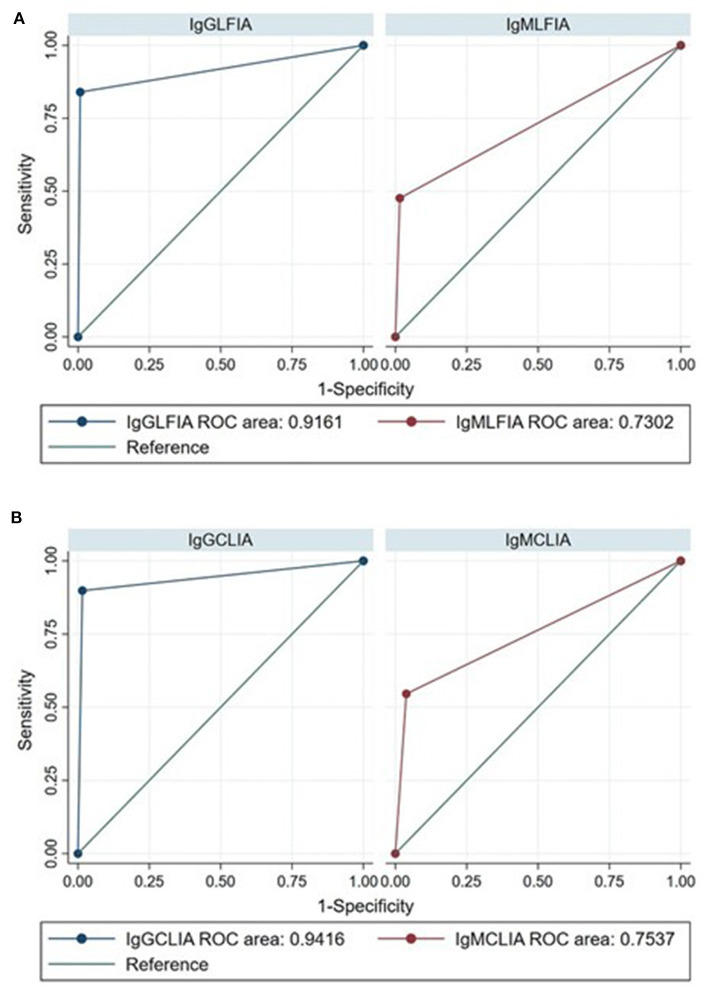
Receiver operating characteristic (ROC) curves for the diagnosis of SARS-CoV-2 infection by IgG and IgM LFIA **(A)** and CLIA-iFlash **(B)** using RT-PCR as reference standard ROC area IgG vs. IgM LFIA, *p* < 0.0001; ROC area IgG vs. IgM CLIA-iFlash, *p* < 0.0001.

As expected, the overall accuracy of IgG serological assays was significantly higher than IgM with both CLIA-iFlash (94.2 vs. 75.4%, *p* < 0.0001) and LFIA (91.6 vs. 73%, *p* < 0.0001) (Figure 1). The ELISA IgA had a sensitivity of 84.3% (95% CI, 78.3–89.2) and specificity of 81.7% (95% CI, 73.1–88.4). However, the result of ELISA IgA was indeterminate in 39 out of 333 (11.7%) individuals, whose 18 out of 203 (8.9%) had RT-PCR positive and 21 out of 130 (16.1%) had RT-PCR negative. If we consider all indeterminate tests as being false negative (in those with RT-PCR positive) or false positive (in those with RT-PCR negative) (worst-case scenario), the sensitivity of ELISA IgA would drop to 76.8% (156/203) and the specificity to 68.4% (89/130).

Table 2 shows the diagnostic performance of serological assays by presence of symptoms. The sensitivity of all tests was lower in asymptomatic than symptomatic individuals, while the specificity was similar. However, in asymptomatic subjects, all IgG serological assays showed a sensitivity around 80%, a part LFIA that yielded a sensitivity of 68.4%.

**Table 2:** Sensitivity and specificity of the serological assays for the diagnosis of SARS-CoV-2 infection in symptomatic and asymptomatic individuals.

**Serological assays**			**No. of** **samples**	**RT-PCR** **positive**	**RT-PCR** **negative**	**True** **positive**	**False** **positive**	**True** **negative**	**False** **negative**	**Sensitivity %** **(95% CI)**	**Specificity %** **(95% CI)**
**Symptomatic individuals**											
IgG	LFIA	POCT	283	187	96	160	1	95	27	85.6 (79.7–90.3)	99.0 (94.3–100)
	CLIA	iFlash	284	188	96	170	2	94	18	90.4 (85.3–94.2)	97.9 (92.7–99.7)
		LIAISON^®^ XL	284	188	96	154	2	94	34	81.9 (75.7–87.1)	97.9 (92.7–99.7)
	ECLIA	Elecsys^®^	284	188	96	164	0	96	24	87.2 (81.6–91.6)	100 (96.2–100)
IgM	LFIA	POCT	283	187	96	94	2	94	93	50.3 (42.9–57.6)	97.9 (92.7–99.7)
	CLIA	iFlash	284	188	96	107	5	91	81	56.9 (49.5–64.1)	94.8 (88.3–98.3)
IgA	ELISA	Euroimmune I	244	168	76	144	19	57	24	85.7 (79.5–90.6)	75.0 (63.7–84.2)
**Asymptomatic individuals**											
IgG	LFIA	POCT	53	19	34	13	0	34	6	68.4 (43.4–87.4)	100 (89.7–100)
	CLIA	iFlash	53	19	34	16	0	34	3	84.2 (60.4–96.6)	100 (89.7–100)
		LIAISON^®^ XL	53	19	34	15	1	33	4	78.9 (54.4–93.9)	97.1 (84.7–99.9)
	ECLIA	Elecsys^®^	53	19	34	15	0	34	4	78.9 (54.4–93.9)	100 (89.7–100)
IgM	LFIA	POCT	53	19	34	4	0	34	15	21.1 (6.0–45.6)	100 (89.7–100)
	CLIA	iFlash	53	19	34	6	0	34	13	31.6 (12.6–56.6)	100 (89.7–100)
IgA	ELISA	Euroimmune I	50	17	33	12	1	32	5	70.6 (44.0–89.7)	97.0 (84.2–99.9)

*RT-PCR, reverse transcriptase-PCR; CI, confidence interval*.

Table 3 shows the sensitivity of serological assays stratified by time from the onset of symptoms. The sensitivity of all serological assays was lower in subjects with onset of symptoms within the 14 days from the blood collection than in those where the onset of symptoms was >14 days. In particular, the sensitivity of LFIA, CLIAs, and ECLIA IgG was 73.8 vs. 91.8%, 80.0 vs. 95.9% (CLIA-iFlash), 70.8 vs. 87.8% (CLIA-LIAISON^®^ XL), and 72.3 vs. 95.1%, in subjects with onset of symptoms within and after 14 days from blood collection, respectively.

**Table 3:** Sensitivity of the serological assays for the diagnosis of SARS-CoV-2 infection by onset of symptoms.

**Serological assays**			**Time elapsed from** **symptoms onset and blood sample collection**					
			**≤14 days**			**>14 days**		
			**No. of** **RT-PCR positive**	**True** **positive**	**Sensitivity** **(95% CI)**	**No.** **RT-PCR positive**	**True** **positive**	**Sensitivity** **(95% CI)**
IgG	LFIA	POCT	65	48	73.8 (61.5–84.0)	122	112	91.8 (85.4–96.0)
	CLIA_S_	iFlash	65	52	80 (68.2–88.9)	123	118	95.9 (90.8–98.7)
		LIAISON^®^ XL	65	46	70.8 (58.2–81.4)	123	108	87.8 (80.7–93.0)
	ECLIA	Elecsys^®^	65	47	72.3 (59.8–82.7)	123	117	95.1 (89.7–98.2)
IgM	LFIA	POCT	65	23	35.4 (23.9–48.2)	122	71	58.2 (48.9–67.1)
	CLIA	iFlash	65	35	53.8 (41–66.3)	123	72	58.5 (49.3–67.3)
IgA	ELISA	EUROIMMUNE I	57	39	68.4 (54.8–80.1)	111	105	94.6 (88.6–98.0)

*RT-PCR, reverse transcriptase-PCR; CI, confidence interval*.

We found a good agreement between the results of the IgG serological assays with *k* values ranging from 0.78 (LFIA vs. CLIA-LIAISON^®^ XL) to 0.94 (LFIA vs. ECLIA-Elecsys^®^), while the agreement was moderate between the IgM assays (*k* = 0.57) (Table 4).

**Table 4:** Agreement between serological assays.

**Serological assays**			**% of agreement**	**Cohen's kappa coefficient**
IgG	LFIA-CLIAs	POCT-iFlash	94.9	0.90
		POCT-LIAISON^®^ XL	88.9	0.78
	LFIA-ECLIA	POCT-Elecsys^®^	97	0.94
	CLIA_S_	iFlash-LIAISON^®^ XL	92.8	0.85
	CLIA_S_-ECLIA	iFlash-Elecsys^®^	96.7	0.93
		LIAISON^®^ XL-Elecsys^®^	91.4	0.82
IgM	LFIA-CLIA	POCT-iFlash	81.2	0.57

Finally, of the 24 patients with RT-PCR negative but considered COVID-19 probable cases, 11 (45.8%) were IgG positive with LFIA, 12 (50%) with CLIA-iFlash, 10 (41.6%) with CLIA-LIAISON^®^ XL, and 11 (45.8%) with ECLIA-Elecsys^®^, while 6 (25%) and 7 (29.2%) were IgM positive with LFIA and CLIA-iFlash, respectively, and 10 (41.6%) with ELISA IgA.

## Discussion

A key aspect for controlling the COVID-19 outbreak is the availability of diagnostic methods that ensure an early and accurate diagnosis of the viral infection (4). To date, few data on serological diagnosis of SARS-CoV-2 infection are currently available (11). In the present study, the diagnostic performances of one point-of-care lateral flow immunochromatographic test and four widely used fully automatic tests for the detection of IgG, IgM, and IgA against SARS-CoV-2 were evaluated by testing plasma samples from subjects with positive and negative SARS-CoV-2 RNA nasopharyngeal swab. The use of a unique and large clinical sample panel to perform the head-to-head comparison of the different serological assays is the strength of our study.

High sensitivities were observed for all four IgG assays, with the CLIA-iFlash resulting to have the highest, with a value equal to 89.9%; the other three assays showed sensitivities not < 80%. Sensitivity stratified by the timing of sample collection in relation to symptoms onset demonstrated that all the IgG assays performed better after 2 weeks from onset of symptoms, with values of sensitivity from 87.8% with CLIA-LIAISON^®^ XL up to 95.9% with CLIA-iFlash. An increase in the IgG-positive rate with time was expected, as IgG are antibodies characteristic of the late stages of infection. Very low values of sensitivity were observed for the two IgM assays that in plasma samples collected after 14 days from the onset of symptoms identified as seropositive approximately half of the RT-PCR SARS-CoV-2 positives. Finally, the sensitivity of ELISA IgA was equal to 84.3% and in symptomatic patients improved overtime (*p* < 0.001), resulting in a sensitivity of 94.6%.

Among the plasma samples from subjects with positive SARS-CoV-2 RNA nasopharyngeal swab about 8% (*n* = 17) was negative by the IgG, IgM, and IgA assays. In particular, three samples were from patients with asymptomatic infection, seven samples were from patients with blood collected during the very early stage of infection (i.e., <7 days after onset symptomatology), and seven plasma samples were from patients with a mean age equal to 83 years. The type of infection, the timing of blood collection, and old age suggest that these patients might have produced low virus-specific antibody levels, not detectable by the serological assays (12–15).

A very good performance in terms of specificity was observed for IgG and IgM assays, with specificities not <97% and equal to 100% by ECLIA-Elecsys^®^ IgG. These findings are in line with those of other studies evaluating SARS-CoV-2 commercial serological methods that reported specificities of IgG and IgM assays ranging from more than 90% up to 100% (14, 16, 17). A lower specificity (81.7%) was observed for ELISA IgA. In addition, according to other authors (18, 19), a significant overall percentage (i.e., 11.7%) of indeterminate results was obtained. The highest indeterminate rate of IgA (i.e., 16.1%) was obtained by testing plasma samples from SARS-CoV-2 RT-PCR–negative cases. Cross-reactivity of ELISA IgA with other respiratory viruses such as influenza A and B and the four common human coronaviruses was reported by some studies (6, 7, 14). We obtained overlapping assays' specificity values by preliminarily investigating a group of 300 archived serum samples collected from healthy blood donors and pregnant women during the pre-pandemic period (i.e., September–October 2019) (data not shown). In this group of samples, the issue of the diagnostic accuracy of the molecular testing in terms of false negatives as well as the possible presence of subjects with a past SARS-CoV-2 infection were overcome.

More variability in sensitivity data was found among studies, particularly for the LFIAs; i.e., sensitivity from 14.4 to 93.1% and from 3 to 69% (95% CI, 60.6–76.3) were reported for the IgG and IgM LFIAs, respectively (14, 20, 21). Sensitivities ranging from 75.4 to 88.9% and equal to 71% were reported for the CLIA IgG and the ECLIA, respectively (14, 16, 17). Sensitivities of 48.1 and 72.1% were reported for the IgM CLIAs (16, 17); finally, sensitivities ranging from 93.3 to 75% were reported for the IgA ELISA (7, 14, 18). It can be hypothesized that this heterogeneity in sensitivity, in addition to the different assays' targets and the problem of the subjective reading of the band in LFIAs (mainly if faint), could also be due to the characteristics of the study populations selected for estimating the assay's sensitivity (Table 5). In fact, variations in the dynamics of the antibody response depending on presence/absence of disease and severity of disease were reported (12, 13). In particular, it was suggested that asymptomatic or paucisymptomatic patients might have low antibody concentrations that could give false-negative results (12). This is in line with our findings given that lower values of sensitivities were observed by investigating plasma from asymptomatic than symptomatic subjects. However, due to the small sample size, no conclusions about the sensitivity performance of the serological assays in asymptomatic population can be drawn from this study. This study has two limitations, i.e., the assays' diagnostic performance was mainly evaluated on symptomatic cases and the case-control study design may have introduced a selection bias. Furthermore, given that a good number of serial serum samples from patients was not available, the kinetics of IgG, IgM, and IgA antibody detection were not analyzed. The results of serological investigations for the diagnosis of SARS-CoV-2 infection have only been examined at two different time-points, during the first 2 weeks and after 14 days after symptoms onset.

**Table 5:** Main characteristics of the studies included in the manuscript.

**Study**	**Study population (number of investigated samples)**		**Clinical** **setting**	**Sample collection** **(days after symptoms onset)**	Serological methods^*^ **(antigens)**
	**Sensitivity assessment**	**Specificity assessment**			
Okba et al. (6)	Confirmed COVID-19 cases (*n* = 41)	Healthy blood donors (*n* = 45); Pts with laboratory-confirmed other virus infection (*n* = 150)	Severe and mild cases	3–27	Commercial and in-house IgG and IgA ELISAs (S, N protein)
Lassaunière et al. (7)	Confirmed COVID-19 cases (*n* = 30)	Healthy individuals before the pandemic (*n* = 10); Pts with laboratory-confirmed other virus infection (*n* = 72)	Inpatients, 100% ICU	7 to >21	Total Ig (S protein), IgG and IgA ELISAs (S protein), IgG-IgM POCTs (not reported)
Charlton et al. (14)	Confirmed COVID-19 cases (*n* = 46)	Healthy individuals before the pandemic (*n* = 50); Pts with laboratory-confirmed other respiratory virus infection (*n* = 62)	Inpatients, 93% (35% ICU); ambulatory, 7%	Mean time: 16; range: 2–48	IgG CMIA (N protein), IgG ECLIA (N protein), IgG CLIA (S1 and S2 domains of S protein), IgG, IgM, and IgA ELISAs (S protein; N protein), IgG-IgM POCTs (not reported; N, S protein)
Infantino et al. (16)	Confirmed COVID-19 cases (*n* = 61)	Healthy individuals before the pandemic (*n* = 20); Pts before the pandemic with rheumatic (*n* = 31) and infectious diseases (*n* = 13)	Inpatients, 100%; 50.8% ICU; 49.2% mild to moderate symptoms	Mean time: 12; range: 8–17	IgG and IgM CLIAs (N, S protein)
Jin et al. (17)	Pts with laboratory-confirmed SARS-CoV-2 (*n* = 43)	Pts with suspected SARS-CoV-2 infection (*n* = 33)	Inpatients, not specified	Median time: 18.0; IQR 11–23	IgG and IgM CLIAs (N, S protein)
Nicol et al. (18)	Pts with laboratory-confirmed SARS-CoV-2 infection (*n* = 141) Pts with probable COVID-19 (*n* = 57)	Pts before the pandemic (*n* = 50); pts with laboratory-confirmed other virus infection (*n* = 25); pregnant women (*n* = 10) and pts with positive rheumatoid factor (*n* = 10)	Majority of pts with symptoms	0 to >15	IgG CLIA (N protein), IgG and IgA ELISAs (S protein), IgG-IgM POCT (N protein)
Van Elslande et al. (19)	Confirmed COVID-19 cases (*n* = 167)	Pts before the pandemic with laboratory-confirmed other respiratory virus infection (*n* = 63) and laboratory-confirmed other virus infection (*n* = 40)	Inpatients, 35% in critical conditions	0–25	IgG and IgA ELISAs (S protein); IgG-IgM POCTs (not reported; N protein)
Imai et al. (20)	Confirmed COVID-19 cases (*n* = 139)	Pts before the pandemic (*n* = 48)	Inpatients, not specified	Median time: 5; IQR, 2–7	IgG-IgM POCT (N, S protein)
Hoffman et al. (21)	Confirmed COVID-19 cases (*n* = 29)	Healthy individuals before the pandemic (*n* = 124)	Pts with symptoms	9–29	IgG-IgM POCT (N, S protein)

* *Commercially available*.

*Pts, patients; ICU, intensive care unit; IQR, interquartile range; NA, not available; S, spike; N, nucleocapsid*.

In addition to the very good analytical performances observed for the IgG assays, a good agreement between the different assay formats was found, with *k* values up to 0.94. Conversely, moderate agreement was observed between the two IgM assays (*k* = 0.57).

Plasma samples from probable COVID-19 cases were also investigated by the five serological assays, and it is noteworthy to mention the seropositivity analysis in this group of patients showed that up to 50% of the cases resulted laboratory confirmed by means of positive serological testing. Larger analyses are advocated given the small sample size here investigated.

In conclusion, the high number of false negatives obtained by the IgM assays seems to limit the use of IgM detection as a marker of acute infection and the high number of indeterminate results obtained by ELISA IgA makes it difficult to clearly define the application area of the search of this class of immunoglobulin. On the other hand, the very good analytical performances in terms of sensitivity, particularly in sera from convalescent phase, and specificity observed for the fully automated high throughput assays for IgG detection, indicate that the search of IgG may represent a reliable tool for epidemiological serosurveys and for retrospective diagnosis of SARS-CoV-2 infection in targeted populations. Moreover, given the ability of the serological assays to detect antibodies in probable COVID-19 group, serological testing could be an important complement to molecular assay for the diagnosis of SARS-CoV-2 infection in this type of patients.

Our findings highlight the potential of serological testing in improving epidemiological control and clinical management of COVID-19. Future studies are certainly required since many questions remain currently unanswered such as the role, pathogenic or protective, of antibody responses during infection, how long antibodies persist after infection, and if the infection results in an immune response that protects individuals from future infections or illness.

## Data Availability Statement

The raw data supporting the conclusions of this article will be made available by the authors, without undue reservation.

## Author Contributions

All authors have accepted responsibility for the entire content of this manuscript and approved its submission.

## Conflict of Interest

The authors declare that the research was conducted in the absence of any commercial or financial relationships that could be construed as a potential conflict of interest.

## Abbreviations

SARS-CoV-2: severe acute respiratory syndrome coronavirus 2
COVID-19: coronavirus disease 2019
RT-PCR: reverse transcriptase-PCR
POC: point-of-care
LFIA: lateral flow immunochromatographic assay
CLIA: chemiluminescence immunoassays
ECLIA: electrochemiluminescence immunoassay
ELISA: enzyme-linked immunosorbent assay
POCT: point-of-care test
TAT: turnaround time
RLU: relative light unit
AU: arbitrary units
EUA: emergency use authorization
COI: cut-off index
CI: confidence interval.
